# The Particular Expression Profiles of Circular RNA in Peripheral Blood of Myocardial Infarction Patients by RNA Sequencing

**DOI:** 10.3389/fcvm.2022.810257

**Published:** 2022-06-06

**Authors:** Qi Li, Yuanyong Wang, Yi An, Jianxun Wang, Yufang Gao

**Affiliations:** ^1^School of Nursing, Qingdao University, Qingdao, China; ^2^Department of Nursing, The Affiliated Hospital of Qingdao University, Qingdao, China; ^3^School of Basic Medical Sciences, Qingdao University, Qingdao, China; ^4^Department of Thoracic Surgery, Tangdu Hospital of Air Force Military Medical University, Xi’an, China; ^5^Department of Cardiology, The Affiliated Hospital of Qingdao University, Qingdao, China; ^6^Department of Emergency, The Affiliated Hospital of Qingdao University, Qingdao, China

**Keywords:** circular RNA, myocardial infarction, RNA sequencing, biomarkers, cardiovascular diseases

## Abstract

Myocardial infarction (MI) is one of the most common illnesses seriously harmful to human health. Notwithstanding, the systems of its pathogenesis are as yet not totally demonstrated. CircRNA is one sort of non-coding RNA, and late distributed information proposes that circRNAs assume a significant part in heart diseases; however, their expression profiles in the peripheral blood of patients with MI are not yet totally characterized. Therefore, RNAs from peripheral blood were recruited for high-throughput RNA-seq analysis. A total of 3,862 circRNAs were distinguished to be remarkably different, including 2,738 circRNAs being upregulated and 1,124 circRNAs being downregulated. circTMEM165, circUBAC2, circZNF609, circANKRD12, and circSLC8A1 were reconfirmed by RT-qPCR in the cell model. ROC curves uncovered that they have great sensitivity and specificity in the determination of MI. Besides, circRNAs are associated with cell metabolism and function by directing complex networks among circular RNAs, microRNAs, and messenger RNAs. In outline, our study portrayed the specific articulation profiles of circular RNA in patients with MI. The outcomes showed that circRNAs might fill in as a sort of ideal biomarkers for MI diagnosis. Further exploration of these circRNAs may enrich our understanding of MI etiology and progression.

## Introduction

Cardiovascular diseases (CVDs) are the most common cause of death globally. The number of deaths from CVD accounted for about one-third of all global deaths (1). Myocardial infarction (MI) is the most common diseases in CVDs and has the highest fatality rate (2, 3). Around 85% of deaths in CVDs are owing to MI and stroke. MI is a multifactorial disease, which involved in complex pathogenesis (4). MI is instigated by partial or complete blockage of the coronary artery (5). The consequence of blocked coronary arteries is an ischemic injury to the region of the heart (6). Once the myocardial cell is damaged by ischemia, dead cardiomyocytes, instead of fibroblasts, ultimately resulted in remodeling, ventricular malfunction, chronic heart failure, and even sudden cardiac death (7–11). It is generally believed that myocardial ischemia is irreversible after 12 h of MI, which is the key factor to impact on patient prognosis (5). Hence, seeking inchoate and accurate biomarkers of MI is urgently needed to improve diagnostic efficiency and the health of the public.

Recently, circRNAs have become the focus and the front field of RNA research based on advanced RNA sequencing technology and bioinformatics analysis. circRNA is a kind of non-coding RNA, which contains a closed continuous loop structure and is broadly expressed in multispecies genomes (12, 13). circRNAs were first revealed years ago, but their functions in cells are freshly appreciating and developing recently (14). Most researchers reported that circRNAs are responsible for regulating gene expression principally by binding with and then resulting in miRNAs dysfunction (9). Ongoing exploration has uncovered that circRNAs work as contending endogenous RNAs or miRNA wipes, as target-RNA decoys by binding to RBPs (15), as regulators of transcription and splicing by binding srnRNA and upgrading Pol II activities (16), and as protein scaffolds (17) and modifiers of parental gene expression. A recent study confirms that circRNAs are a key factor in CVDs (18). Abnormally expressed circRNAs were strongly linked to the occurrence and progress of CVDs, for instance, MI (9), hypertrophy, heart failure (19), and atherosclerosis (20), indicating the potential effects of circRNA in CVDs. Taken together, a lot of evidence affords a novel orientation for discovering circRNAs as a new-fashioned biomarker for the diagnosis of CVDs.

The peripheral blood involves numerous leukocyte subpopulations, for instance, T cells, B cells, mononuclear cells, and natural killer cells. Studies have shown that key genes in leukocytes are associated with alterations in gene expression and the onset of disease (21).

To date, the associations between circRNAs in peripheral blood and MI are not fully understood. Therefore, we explored the expression profiles of circRNA in patients with MI and healthy controls by high-throughput circRNA sequencing. We collected the volunteers’ peripheral blood and constructed the cell model to confirm the dysregulated circRNAs. ROC and combined ROC curves were created for demonstrating the biomarkers of MI. The roles of circRNAs in MI were analyzed by bioinformatics. Moreover, we discovered a possible complex endogenic RNA regulatory network among circRNAs-miRNAs-mRNAs, with a view to providing some references for related research in this field.

## Materials and Methods

All testing programs were performed according to the protocols approved by the Medical Ethics Committee of the Affiliated Hospital of Qingdao University. All sample providers were informed of their blood samples, clinical data were used for this study, and informed consents were signed.

### Sample Collection

The peripheral blood samples from 80 patients with MI were collected before percutaneous coronary intervention surgery between March 2018 and January 2019 in the Affiliated Hospital of Qingdao University (19). All patients were not treated with heparin, radiotherapy, or chemotherapy. The matched healthy controls were collected from the volunteers of the physical examination center (Supplementary Table 4). Fresh peripheral blood samples (5 ml) from patients with MI and healthy individuals were collected in EDTA tubes.

### Total RNA Extraction

The samples were used for RNA extraction *via* the TRIzol method (Trizol-up reagents, TransGen, China). This experiment was performed according to the kit’s recommendations. RNA samples were stored at −80°C.

### RNA Quantification and Qualification

The purity of the extracted RNA was tested by a NanoPhotometer^®^ Spectrophotometer (IMPLEN, CA, United States). RNA concentration was detected by Qubit^®^ RNA Assay Kit in Qubit^®^ 2.0 Flurometer (Life Technologies, CA, United States). RNA integrity was measured by the RNA Nano 6000 Assay Kit of the Bioanalyzer 2100 system (Agilent Technologies, CA, United States). RNA degradation and contamination were monitored on 1% agarose gels.

### Library Preparation and circRNA Sequencing

A total of 5 μg RNA per sample was used for the RNA library preparations. First, rRNA was removed by Epicenter Ribozero*™* rRNA Removal Kit (Epicenter, United States) and ethanol precipitation. Subsequently, the linear RNA was digested with RNaseR (Epicenter, United States). The sequencing libraries were produced by NEBNext^®^ Ultra*™* Directional RNA Library Prep Kit for Illumina^®^ (NEB, United States).

The cluster generation of the index-coded samples was performed on a cBot Cluster Generation System using TruSeq PE Cluster Kit v3-cBot-HS. After that, the libraries were sequenced on an Illumina Hiseq 4000 platform. These experiments were performed following the manufacturer’s instructions.

### Data Analysis and circRNA Identification

First, clean data were gained by wiping off reads containing adapter and low-quality reads from raw data. The clean data with high quality were used for downstream analyses. Index of the reference genome was created using Bowtie2 v2.2.8, and paired-end clean reads were united to the reference genome using Bowtie (22). The circRNAs were detected and verified by using find_circ (23) and CIRI2 (24). The Circos software was used to construct the circos figure.

### Divergent Polymerase Chain Reaction

The junction site part of circRNAs was confirmed by polymerase chain reaction (PCR) with divergent primers. Convergent primers were used as the control. The products of the PCR amplification were authenticated by 1% agarose gel electrophoresis. The primers for circRNA validation are listed in Supplementary Table 1.

### Real-Time Quantitative Polymerase Chain Reaction

The dysregulation circRNA was reconfirmed on a CFX96 Real-Time PCR Detection System (Bio-Rad). Total RNA was extracted using Trizol reagent. Reverse transcription reactions were performed by using the TransScript II One-Step gDNA Removal and cDNA Synthesis SuperMix (TransGen, China) to make cDNA according to the manufacturer’s guide. TransStart Green qPCR SuperMix (TransGen) was used for quantitative PCR (qPCR) analysis, and the procedures were performed in accordance with the kit instructions. The levels of dysregulation circRNA analyzed by RT-qPCR were normalized to that of GAPDH (21). The primers for RT-qPCR are listed in Supplementary Table 2.

### Creation of Receiver Operating Characteristic Curves

The ROC curves were created to estimate the diagnostic value of circRNAs for MI. An ROC curve was calculated, and the specificity and sensitivity of predictive power were measured by the AUC. The AUC was used to assess the diagnostic value of the circRNAs in the peripheral blood of patients with MI (25). The combination ROC is made by the combined predictors obtained by the regression of the five genes of interest. SPSS26.0 and Graphpad prism7 were used for calculating and drawing the ROC curve.

### Gene Ontology and Kyoto Encyclopedia of Genes and Genomes Enrichment Analysis

Gene Ontology (GO) enrichment analysis for host genes of differentially expressed circRNAs was carried out by the GO seq R package, and the gene length bias was corrected during this procedure (26). GO terms with corrected *P*-value less than 0.05 were considered to be significantly enriched by differential expressed genes. As a database resource for understanding high-level functions and utilities of the biological system (27), Kyoto Encyclopedia of Genes and Genomes (KEGG) is used to explore information from the molecular level (such as the cell, the organism, and the ecosystem), especially large-scale molecular datasets generated by genome sequencing and other high-throughput experimental technologies.^1^ We used the KOBAS software to test the statistical enrichment of differential expression genes or circRNA host genes in KEGG pathways (28).

### Prediction of the Network of circRNAs-MiRNAs-mRNAs

circRNA can regulate the translation of mRNA *via* binding to an miRNA (9). miRNA target sites in the exons of circRNA loci were verified by using miRanda. The binding sites of miRNA and mRNA were predicted by RNAhybrid. Cytoscape was used to construct the circRNA-miRNA-mRNA networks.

### Cell Culture and Treatment

AC16 cells were cultured in DMEM (Gibco) with 10% fetal bovine serum (TransGen), and 100 U/ml penicillin and 100 mg/ml streptomycin (Invitrogen) in a humidified 5% CO_2_ incubator at 37°C. When the cultured cells reached approximately 70% confluently, they were treated with H_2_O_2_ (100 μM and 500 μM, respectively) incubated at 37°C for 12 h in a complete culture medium. The cells were collected for RNA extraction and dysregulated circRNA reconfirmation.

### Apoptosis Assays

Apoptosis was determined by the terminal deoxynucleotidyl transferase-mediated dUTP nick-end labeling (TUNEL) using a kit from TransGen. The detection procedures were in accordance with the kit instructions.

### Mitochondrial Staining and Analysis of Mitochondrial Fission

Mitochondrial staining was performed as described earlier with modifications (29). Briefly, cells were plated onto the poly-L-lysine-coated coverslips. After treatment, they were stained for 30 min with 0.02 μM MitoTracker Red at 37°C. Mitochondria were imaged using a laser-scanning confocal microscope (Zeiss LSM510 META).

### Immunoblotting

Immunoblot was carried out as described earlier (30). Briefly, the cells were lysed for 20 min on ice in RIPA lysis buffer containing a protease inhibitor cocktail and DMSF. The samples were subjected to 12% SDS-PAGE and transferred to nitrocellulose membranes. Blots were probed with primary antibodies anti-Caspase-3 (Abcom, 1:1,000), anti-Cyto c (Abclone, 1:1,000), anti-Tublin (Affinity, 2:1,000), and anti-β-actin (TransGen, 1:2,000) at 4°C overnight with gently shaking. After three times washing with PBS, the horseradish peroxidase (HRP)-conjugated secondary antibodies were added. Antigen-antibody complexes were visualized by enhanced chemiluminescence. Enhanced ECL TM prime detection reagent (30) was used to visualize antigen-antibody complexes, and the density was quantified by ImageJ.

### Statistical Analysis

The results are expressed as mean ± SEM of at least three independent experiments. The statistical comparison between different groups was completed by one-way ANOVA for multiple comparisons or *t*-test with Welch’s correction for two groups. Statistical analyses were completed with GraphPad Prism 7.0 (GraphPad Software, Inc., San Diego, CA). *P* < 0.05 was regarded as statistically significant.

## Results

### The Landscape of circRNA Expression Profiles in Peripheral Blood From Patients With Myocardial Infarction and Healthy Individuals

**FIGURE 1:**
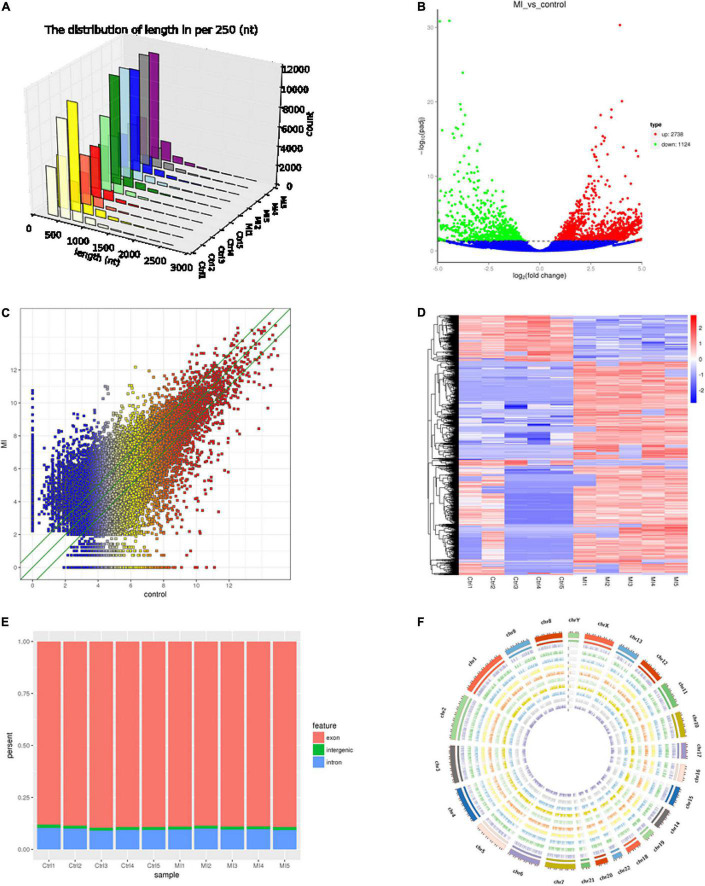
Landscape of circRNA profiles. **(A)** Length distribution of circRNAs (29). **(B)** Volcano plot of differentially expressed circRNAs in MI. The abscissa represents the fold change of circRNA in different groups, and the ordinate represents the level of statistical significance. The plots in the figure represent circRNAs. The blue points denote no significant difference, red points denote that the expression levels of circRNAs are significantly upregulated, and green points denote that the expression levels of circRNAs are significantly downregulated (31). **(C)** The scatter plot presents the circRNA expression variations between the MI and paired healthy controls. The nodal increment of the x and y axes is 1 (log2FC = 1). The circRNAs above the top green line and below the bottom green line indicate greater than 2-fold change (TPM > 2) between the MI and control. **(D)** Hierarchical cluster analysis shows the differential expression profiles of circRNAs among MI samples and normal samples. The upregulated and downregulated circRNAs are colored in red and blue, respectively. Ctrl1-Ctrl5 represent healthy controls, and MI1-MI5 represent MI patients. **(E)** Histogram shows the percentage of circRNA from different genomic origins. **(F)** Pie chart shows the differentially expressed circRNAs based on chromosomal distributions.

The circRNAs were identified by find_circ and Bowtie2 based on the high-quality clean data. A total of 3,862 circRNAs were recognized in five pairs of MI and healthy controls samples. The size of the circRNAs extended from ≈100 to 2,500 nt (Figure 1A). The differentially expressed circRNAs were analyzed by DEGseq, and the results were considered statistically significant when padj < 0.05. The differentially expressed profiles of circRNAs among the two groups are shown in Figures 1B,C. Hierarchical clustering heatmap analysis shown in Figure 1D illustrates a unique circRNA expression landscape among the MI and healthy individuals. Our results presented that there was an obvious difference in circRNA expression profile between the MI and healthy controls. Consequently, 3,862 circRNAs were altered in the patients with MI. In total, 2,738 circRNAs were upregulated and 1,124 circRNAs were downregulated in the MI group compared with the healthy controls. Of the distinguishably expressed circRNAs, 89% are exonic, 10% are intronic, and 1% are intergenic (Figure 1E). As shown in Figure 1F, the transcription of dysregulated circRNAs was observed to be broadly dispersed in all chromosomes, and chr1, chr2, chr3, chr4, chr5, chr6, chr7, chr8, chrX, and chr9 are the top ten circRNAs. The top 50 differentially expressed circRNAs are depicted in Supplementary Table 2.

### CircRNAs Screening and Circle Structure Identification

**FIGURE 2:**
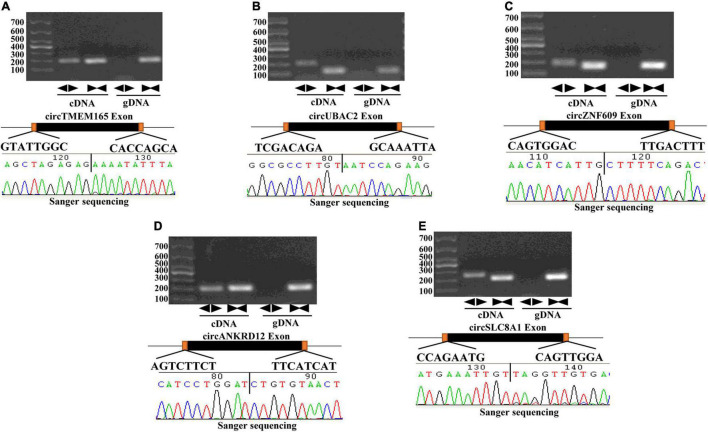
Validation of the circular structure of circRNA. circRNAs exist in human myocardial cell line (AC16). Clear single bands were amplified from the cDNA of AC16 by divergent primers, while they could not be amplified from gDNA. Sanger-Seq validated the head-to-tail junction of circRNAs. circTMEM165 **(A)**, circUBAC2 **(B)**, circZNF609 **(C)**, circANKRD12 **(D)**, circSLC8A1 **(E)** exists in human myocardial cell.

First, we screened candidate circRNAs by comparing our results with the previous study on human heart tissue sequencing sample results (Supplementary Figure 1) (31) in order to improve the accuracy of our research. Then, we detected the circle structure of intersection circRNAs. We tested the expression of intersection circRNAs by adding them to the cDNA of myocardial cells by using the divergent primer (Supplementary Table 1). Additionally, the added products of circRNAs were sequenced by using Sanger sequencing to further confirm the circularized junction size of the circRNAs (Figure 2).

### Authentication of Differentially Expressed circRNAs

**FIGURE 3:**
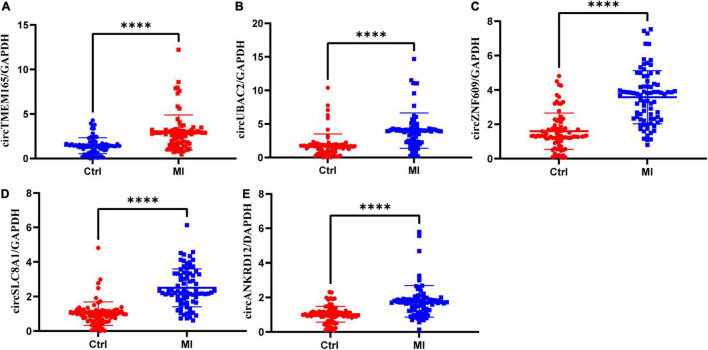
Validation of differentially expressed circRNAs. RT-qPCR validation of selected circRNAs. **(A–E)** The relative expression levels of circRNAs in peripheral blood MI patients and controls (Ctrl: healthy controls; MI: myocardial infarction patients; ^***^P < 0.0001).

To verify the RNA-seq data, five intersectional circRNAs (i.e., circTMEM165, circUBAC2, circZNF609, circANKRD12, and circSLC8A1) were selected and validated by RT-qPCR using 80 MI samples and 80 healthy individuals. The general information of selected circRNAs shown as Table 1. The results revealed that the expression of these five circRNAs was upregulated in patients with MI (Figure 3). The RT-qPCR data were strongly consistent with the circRNA sequencing results.

**TABLE 1:** The general information of circRNA.

circBase_ID	Gene name	MI_readcount	Control_readcount
hsa_circ_0000826	ANKRD12	816.599493	79.5731602
hsa_circ_0001414	TMEM165	720.396765	102.724399
hsa_circ_0000615	ZNF609	509.23034	0
hsa_circ_0030720	UBAC2	328.578223	24.0160633
hsa_circ_0000994	SLC8A1	313.178477	0
			

**FIGURE 4:**
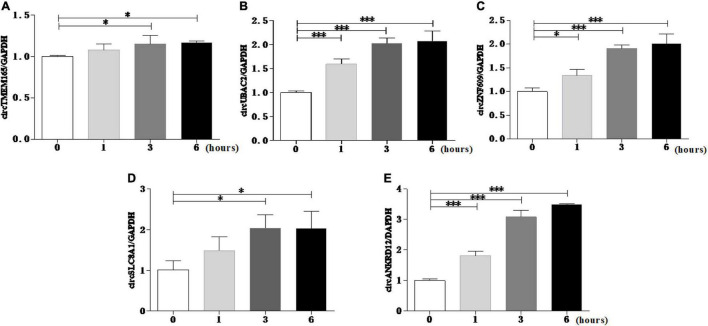
Reconrmation of differentially expressed circRNAs in cell model. Myocardial cell injury model was used to simulate cell damage during MI, and the expression level of circTMEM165 **(A)**, circUBAC2 **(B)**, circZNF609 **(C)**, circSLC8A1 **(D)**, circANKRD12 **(E)** were analyzed by RT-qPCR (*P < 0.05, ^***^P < 0.0001).

To reconfirm the dysregulation of these circRNAs in the cell model, we used the myocardial cell injury model induced by reactive oxygen species (ROS) to simulate cell damage during MI (9). The data of the cell model showed that all the validated circRNAs were upregulated with prolonged stimulation time (Figure 4). This result was consistent with the data on peripheral blood. Collectively, these two results verified the accuracy and repeatability of the circRNA sequencing results.

### The Potential Diagnostic Values of circRNAs for Patients With Myocardial Infarction

**FIGURE 5:**
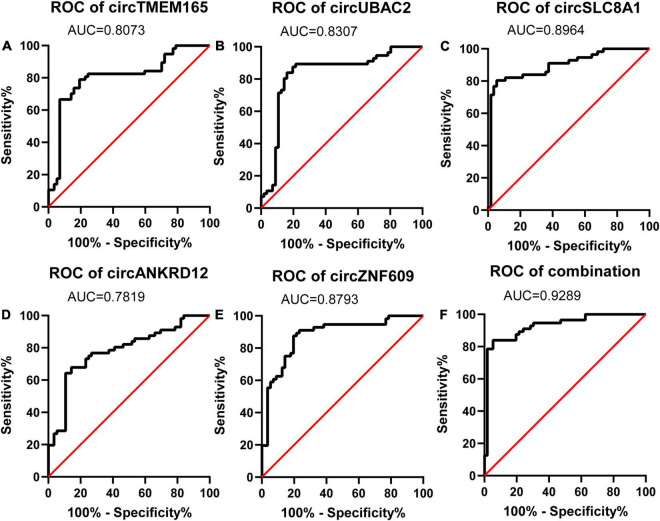
Diagnostic value of circRNAs validated by RT-qPCR. Analysis of the sensitivity and specificity of circRNAs and circRNAs combination as a novel MI marker by ROC curve. **(A–E)** ROC curves of five RT-qPCR-validated circRNAs. **(F)** ROC curves of circRNAs combination (*n* = 80).

To evaluate the potential diagnostic value of selected circRNAs for MI, we computed and created ROC curves (Figure 5) for RT-qPCR-validated circRNAs to discriminate patients with MI from healthy individuals according to the fold changes of these circRNAs’ expression. We trained the model with 70% of the samples and validated it with the remaining 30% of the data (Supplementary Figure 2).

The calculated optimal cutoff point for circTMEM165 level in the peripheral blood was 1.6089505 (Youden index = 0.55), while the sensitivity and specificity were 21.3% and 23.7%, respectively. The calculated optimal cutoff point for circUBAC2 level was 1.968652 (Youden index = 0.663), while the sensitivity and specificity were 82.5% and 83.8%, respectively. The calculated optimal cutoff point for circ ZNF609 level was 1.947015 (Youden index = 0.638), while the sensitivity and specificity were 86.3% and 77.5%, respectively. The calculated optimal cutoff point for circ SLC8A1 level was 1.5414885 (Youden index = 0.737), while the sensitivity and specificity were 82.5% and 91.25%, respectively. The calculated optimal cutoff point for circANKRD12 level was 1.4872795 (Youden index = 0.575), while the sensitivity and specificity were 87.5% and 57.5%, respectively. The optimal cutoff point of the combination was 0.06, while the sensitivity, specificity, and Youden index were 0.863, 0.937, and 0.8, respectively. The results revealed that the AUC of these circRNAs at least reached 0.8073, and the AUC of recombination circRNAs reached up to 0.9289 (Table 2), indicating that these circRNAs expressed in peripheral blood could set apart the patients with MI from the healthy individuals. Our data discovered the potential values of circRNAs MI diagnosis.

**TABLE 2:** AUC of RT-qPCR-validated circRNAs in patients’ validation.

circRNA	Validation patients (*n* = 50)		
	AUC	95% Confidence interval	*P*value
circTMEM165	0.8073	0.7225 to 0.8921	< 0.0001
circUBAC2	0.8307	0.7466 to 0.9147	< 0.0001
circZNF609	0.8793	0.8136 to 0.9450	< 0.0001
circSLC8A1	0.8964	0.8345 to 0.9583	< 0.0001
circANKRD12	0.7819	0.6950 to 0.8688	< 0.0001
circRNAs combination	0.9289	0.8793 to 0.9784	< 0.0001

### Enrichment Analysis of circRNAs Host Genes

**FIGURE 6:**
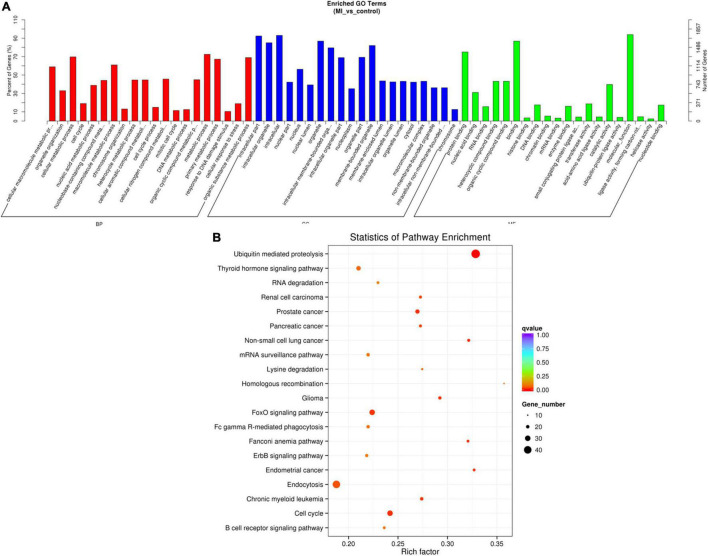
GO and KEGG of differentially expressed circRNA genes. **(A)** GO analysis of differentially expressed circRNAs which covers 3 domains, namely, BP (red), CC (blue), and MF (green). Each domain contains the top 20 significantly enriched GO terms (29) and the top 20 significantly enriched pathways. **(B)** KEGG The y axis denotes the pathway, and the x axis denotes the rich factor. The size of the dots indicates the number of gene regulation in this pathway, and the colors of the dots correspond to different *q*-values. BP: biological process, CC: cellular component, MF: molecular function.

The source genes of the validated differentially expressed circRNAs were uncovered by using GO annotation, as shown in Figure 6A. The most remarkably improved GO terms about the biological process (BP), cellular component (CC), and molecular function (MF) classes were cellular macromolecule metabolic process (GO:0044260, *P* = 2.892E-46), intracellular part (GO:0044424, *P* = 5.136E-69), and protein binding (GO:0005515, *P* = 8.9672E-34), respectively.

The data of the KEGG pathway study of the circRNAs genes are shown in Figure 6B. The most relevant genes of differentially expressed circRNAs are ubiquitin-mediated proteolysis (rich factor = 0.328467153, q-value = 1.56E-06, gene number = 45).

### Predicted circRNA-miRNA-mRNA Regulatory Network

**FIGURE 7:**
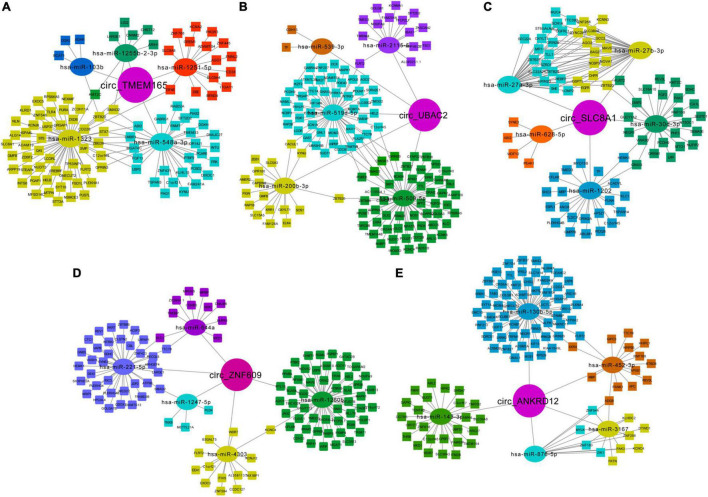
Predicted network of circRNA-miRNA-mRNA. Predicted circRNA-miRNA-mRNA network for the five upregulated circRNAs in MI: **(A)** circ-TMEM165 (29), **(B)** circ-UBAC2 (31), **(C)** circ-SLC8A1, **(D)** circ-ZNF609, and **(E)** circ-ANKRD12. Circle represents circRNAs, oval represents miRNAs, and rectangle represents mRNAs.

The miRNA binding sites exist in circRNAs, which can function as miRNA sponges. To further explore their possible roles in patients with MI, we predicted the circRNA-miRNA-mRNA network and analyzed using miRanda and RNAhybrid. The top five predicted miRNAs for these upregulated circRNAs are shown in Table 3, and the target mRNAs of miRNAs are listed in Supplementary Table 3. Additionally, circRNA-miRNA-targeted mRNA networks are also constructed for showing regulatory relationships between them (Figure 7).

**TABLE 3:** The predicted miRNAs related to circRNAs.

Upregulated circRNAs	Predicted miRNAs
circTMEM165	hsa-miR-103b
	hsa-miR-1255b-2-3p
	hsa-miR-1323
	hsa-miR-548a-3p
	hsa-miR-1251-5p
circUBAC2	hsa-miR-200b-3p
	hsa-miR-2115-5p
	hsa-miR-509-5p
	hsa-miR-519d-5p
	hsa-miR-539-3p
circZNF609	hsa-miR-644a
	hsa-miR-4303
	hsa-miR-221-5p
	hsa-miR-1260b
	hsa-miR-1247-5p
circSLC8A1	hsa-miR-1202
	hsa-miR-27a-3p
	hsa-miR-27b-3p
	hsa-miR-30d-3p
	hsa-miR-628-5p
circANKRD12	hsa-miR-876-5p
	hsa-miR-452-3p
	hsa-miR-3167
	hsa-miR-143-3p
	hsa-miR-130b-5p

Moreover, we noted the potential function of these regulatory networks. For example, circTMEM165 is predicted as “sponge” to hsa-miR-548a-3p, which can bind to mRNA GREM1, which is responsible for regulating organogenesis, body patterning, and tissue differentiation. The network of circZNF609-hsa-miR-221-5p-WDR7 may be involved in the low-density lipoprotein (LDL) metabolism. The regulatory pathway of circANKRD12-hsa-miR-876-5p-MYLK may promote myosin interaction with actin and facilitate to produce contractile activity. circANKRD12-hsa-miR-130b-5p targets both ACSM2A and RNF213, which regulate mitochondrial acyl-coenzyme A synthetase and ATPase activity, respectively. These networks indicate the possible interactional RNA regulatory relationships between circRNAs, miRNAs, and mRNAs in MI.

### Knockdown circSLC8A1 Reduced Myocardial Cell to Apoptosis Under Oxidative Stress

**FIGURE 8:**
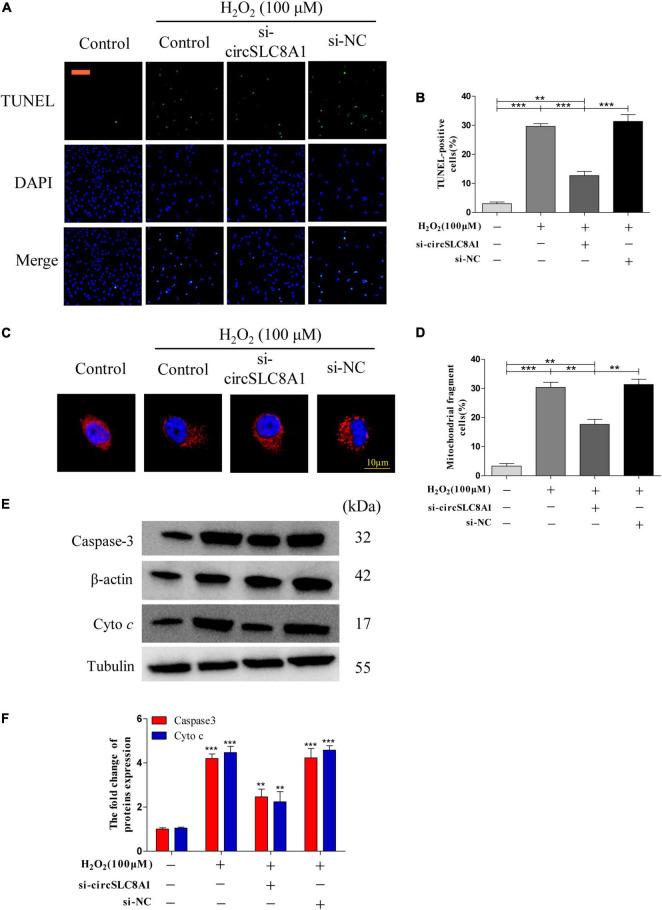
Knockdown circSLC8A1 reduced myocardial cell to apoptosis under oxidative stress. **(A)** AC16 cells were exposed to 100 μM H_2_O_2_ for another 12 h after transfected with circSLC8A1-siRNA for 24 h, and apoptosis was stained with TUNEL. **(B)** The percentage of cells underwent TUNEL-positive cell. **(C)** AC16 cells were exposed to 100 μM H_2_O_2_ for another 12 h after the knockdown of circSLC8A1 expression, and the mitochondria was stained with MitoTracker Red. **(D)** The percentage of cells underwent mitochondrial fission. **(E)** The expression levels of apoptotic-related proteins were detected by western blotting. **(F)** The data analysis of blot densitometry. All the data were expressed as the mean ± SEM of three independent experiments. *P < 0.05, **P < 0.01, ***P < 0.001.

As circSLC8A1 has the best ROC in the verification of clinical samples, we should further explore its mechanism of action in MI. To further clarify the underlying mechanism of circSLC8A1 in MI, we first knocked down circSLC8A1 expression in the myocardial cells through circSLC8A1-siRNA. The AC16 cells were treated with 100 μM H_2_O_2_ after the knockdown of circSLC8A1 expression. We found that the number of apoptotic cells was significantly decreased in the si-circSLC8A1 group (Figures 8A,B), and the percentage of fragmented mitochondrial cells were significantly reduced in the siRNA group compared with other groups (Figures 8C,D). To investigate whether circSLC8A1 is involved in mitochondrial apoptosis, we detected the expression level of apoptosis-related proteins *via* Western blot analysis, and our results revealed that the expression levels of Caspase3 and cyto c were markedly decreased (Figures 8E,F). These data revealed that under slight oxidative stress, cell apoptosis was more hard induced after the knockdown of circSLC8A1.

Taken together, these data demonstrated that knockdown of circSLC8A1 decreased intracellular oxidative stress, kept the balance of mitochondria dynamics, and then reduced apoptosis during oxidative stress.

### Overexpression of circSLC8A1 Sensitized Myocardial Cell to Apoptosis Under Oxidative Stress

**FIGURE 9:**
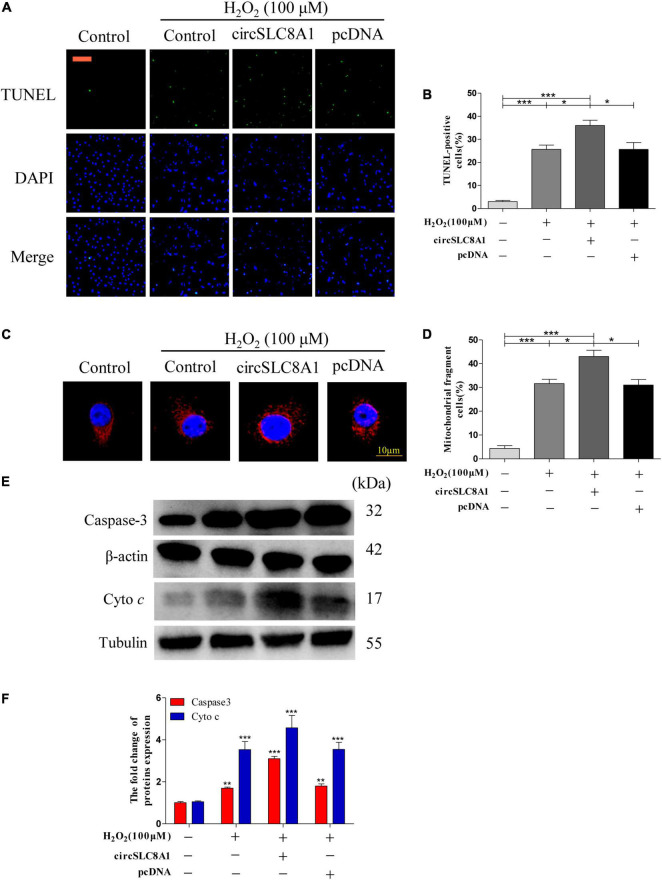
Overexpression of circSLC8A1 sensitized myocardial cell to apoptosis under oxidative stress. **(A)** AC16 cells were exposed to 100 μM H_2_O_2_ for another 12 h after transfected with circSLC8A1-cDNA for 24 h, and apoptosis was stained with TUNEL. **(B)** The percentage of cells underwent TUNEL positive cell. **(C)** AC16 cells were exposed to 100 μM H_2_O_2_ for another 12 h after the overexpression of circSLC8A1, and the mitochondria was stained with MitoTracker Red. **(D)** The percentage of cells underwent mitochondrial fission. **(E)** The expression levels of apoptotic-related proteins were detected by western blotting. **(F)** The data analysis of blot densitometry. All of the data were expressed as the mean ± SEM of three independent experiments. *P < 0.05, **P < 0.01, ***P < 0.001.

To understand the role of circSLC8A1 in myocardial cell apoptosis, we overexpressed circSLC8A1 in AC16 cells using circSLC8A1-plasmid. After transfection, the cell was treated with 100 μM H_2_O_2_. The results indicated that overexpression of circSLC8A1 remarkably promotes mitochondria fission (Figure 9C), and the percentage of fragmented mitochondrial cells were significantly increased (Figure 9D). In cell apoptosis assays, we found that the proportion of TUNEL-positive cells was raised compared with the related controls (Figures 9A,B). We also examined the expression level of apoptosis-associated proteins by Western blot assay; the results showed that in the circSLC8A1 OE group, the expression levels of Caspase3 and cyto c were significantly increased (Figures 9E,F).

Altogether, our results demonstrated that the overexpression of circSLC8A1 increased the intracellular oxidative stress, and it sensitized the myocardial cells to apoptosis by further increasing mitochondrial fission under the stimulation of oxidative stress.

## Discussion

Myocardial infarction is a kind of multifactorial disease, which causes complex pathogenesis and leads to high mortality. It is hard to diagnose and prompt the intervention of MI before the disease occurs. As a consequence, there is an earnest for early detection markers for MI diagnosis. circRNAs are single-stranded, covalently closed molecules (32) that are expressed in numerous species (23, 33). The application of cutting-edge RNA-seq innovation has improved our understanding of knowledge about the specific circRNA expression profiles in human diseases (12). Some researchers pointed out that circRNAs may serve as a novel kind of ideal biomarker for disease diagnosis (34). In this study, circRNA-seq was employed to distinguish differences in the expression profiles of circRNA in peripheral blood between patients with MI and healthy individuals, and our results revealed the possible contribution of differentially expressed circRNAs in MI pathology.

Recent reports have demonstrated that circRNAs are more constant, varied, and conserved when compared with other kinds of RNAs, including mRNAs, miRNAs, and lncRNAs (23). circRNAs are extensively expressed in multi-species (23, 33) and are involved in disease occurrence. These characteristics of circRNA might endow them to be a new kind of biomarker for disease diagnosis. To discover the novel and original biomarkers of MI, we first verified the expression landscape of circRNAs in human peripheral blood between five patients with MI and five healthy individuals by using RNA-seq. A total of 3,862 circRNAs were significantly differentially expressed between MI and control groups. According to the screening conditions, we finally selected and verified 5 circRNAs (i.e., circTMEM165, circUBAC2, circZNF609, circANKRD12, and circSLC8A1). With further large clinical sample reconfirmation by RT-qPCR, the results strongly consisted with the sequencing data.

Early diagnosis can guide clinical medication to effectively treat MI and significantly improve their prognosis. circRNAs have already been confirmed as effective biomarkers for numerous diseases, such as cancer (35), acquired pneumonia (36), and Kawasaki disease (37). It is reported that hsa_circ_0004104 was upregulated in coronary artery disease (CAD), and its overexpression might contribute to the pathogenesis of CAD (38). Hence, circRNA also should improve the accuracy of diagnosis of MI. In this study, our data revealed that thousands of circRNAs were observably dysregulated in the peripheral blood from patients with MI, and circTMEM165, circUBAC2, circZNF609, circANKRD12, and circSLC8A1 were selected for further study. As expected, these five circRNAs were reconfirmed as being markedly upregulated in the peripheral blood of 80 patients with MI by RT-qPCR. The AUCs of these five circRNAs were 0.8073, 0.8307, 0.8793, 0.8964, and 0.7819, respectively. When we analyzed these five circRNAs in a combined manner, the AUC was even up to 0.9289. Our data indicated that these circRNAs might be significantly associated with MI and have the potential to assist in the diagnosis of MI.

Recently, several studies have indicated that circRNAs might be key regulators in CVDs. For instance, Wu et al. (37) showed that circANRIL and hsa_circ_0123996 levels in the serum of patients with Kawasaki disease were markedly different from those in healthy controls, and the expression level will be changed after therapy. Wang et al. (38) found that Hsa_circ_0001879 and hsa_circ_0004104 were confirmed to be markedly upregulated in patients with CAD, and the overexpression of hsa_circ_0004104 contributed to the dysregulation of atherosclerosis-related genes. All these findings powerfully support the view that circRNAs play crucial roles in the occurrence and progression of CVDs.

We also used circRNA-seq data to predict circRNA-miRNA-mRNA networks and aimed to find the possible roles of circTMEM165, circUBAC2, circZNF609, circANKRD12, and circSLC8A1 in the peripheral blood of patients with MI. We observed the possible target mRNAs of these circRNAs including GREM1, WDR7, MYLK, ACSM2A, and RNF213. Moreover, the predicted target mRNAs played a key role in regulating organogenesis, body patterning, tissue differentiation, LDL metabolism, and myosin interaction with actin, mitochondrial acyl-coenzyme A synthetase, and ATPase activity, respectively. All of them are highly correlated with CVDs.

To further clarify the potential mechanism of circSLC8A1 in MI, we tested the cell function through knockdown and overexpressed circSLC8A1 based on the cell model. Uncontrolled mitochondrial fission contributes to apoptosis (39), which promotes the development of heart injury. Our results indicated that the knockdown of circSLC8A1 could maintain mitochondrial homeostasis and hence prevent the occurrence of apoptosis. Further experimental studies are needed to prove the specific signaling pathway.

A few limitations of this study ought to be mentioned. First, we only verified 10 circRNAs that were upregulated in MI, and still, a lot of differentially expressed circRNA needed to be verified. Second, although we use a set of filter methods to get the more accurate results, the sample size of this study is still limited, which will increase the false positive rate while analyzing RNASeq data. Third, we only explore more upregulated circRNAs, and the downregulated circRNAs are also worth studying in depth. Moreover, further experiments are needed to explore the regulatory effects of circRNAs in the occurrence of MI in patients with CVD.

## Conclusion

To the best of our knowledge, this study is the first to report the expression profiles of circular RNA in the peripheral blood of patients with MI compared with healthy individuals. Bioinformatics analysis indicated that the upregulated expressions of circTMEM165, circUBAC2, circZNF609, circANKRD12, and circSLC8A1 were highly correlated with MI. Moreover, all of these five circRNAs may be used to improve the diagnosis accuracy of MI. Besides, the bioinformatics analysis found that these circRNAs could be involved in the regulation of hundreds of mRNAs, and numerous of these mRNAs may affect the occurrence of MI. Although our results revealed that these circRNAs are highly expressed in the peripheral blood of patients with MI, their predicted functions still need to be confirmed by further experiments.

## Data Availability Statement

The original contributions presented in the study are included in the article/Supplementary Material, further inquiries can be directed to the corresponding authors.

## Ethics Statement

The studies involving human participants were reviewed and approved by Ethics Committee of Affiliated Hospital of Qingdao University. The patients/participants provided their written informed consent to participate in this study.

## Author Contributions

QL, JW, and YG conceived and designed the experiments. QL, YW, and YA collected and analyzed the data. QL and YW wrote this manuscript. All authors read and approved the final manuscript.

## Conflict of Interest

The authors declare that the research was conducted in the absence of any commercial or financial relationships that could be construed as a potential conflict of interest.

## Publisher’s Note

All claims expressed in this article are solely those of the authors and do not necessarily represent those of their affiliated organizations, or those of the publisher, the editors and the reviewers. Any product that may be evaluated in this article, or claim that may be made by its manufacturer, is not guaranteed or endorsed by the publisher.

## Footnotes

1
http://www.genome.jp/kegg/

## Supplementary Material

Supplementary Figure 1 circRNA screening. Totally 5 circRNAs were screened out after comparison of our results with human heart tissue sequencing results.

Supplementary Figure 2 ROC curve validation; 30% of the random sample was used to verify the ROC curve reliability of our data trained.

## Abbreviations

MI: myocardial infarction
circRNA: circular RNA
RT-qPCR: real-time quantitative polymerase chain reaction
ROC: receiver operating characteristic
GO: Gene Ontology
KEGG: Kyoto Encyclopedia of Genes and Genomes
miRNA: microRNA
CVDs: cardiovascular diseases
RNA-Seq: high-throughput RNA sequencing
EDTA: ethylenediaminetetraacetic acid
qPCR: quantitative PCR
AUC: the area under the curve
ROS: reactive oxygen species
BP: biological process
CC: cellular component
MF: molecular function
LDL: low-density lipoprotein
CAD: artery disease.
