# Supplementary figures and images for: The Particular Expression Profiles of Circular RNA in Peripheral Blood of Myocardial Infarction Patients by RNA Sequencing
Source: Front Cardiovasc Med. 2022 Jun 6;9:810257. doi: 10.3389/fcvm.2022.810257 (PMC9207279; doi:10.3389/fcvm.2022.810257)

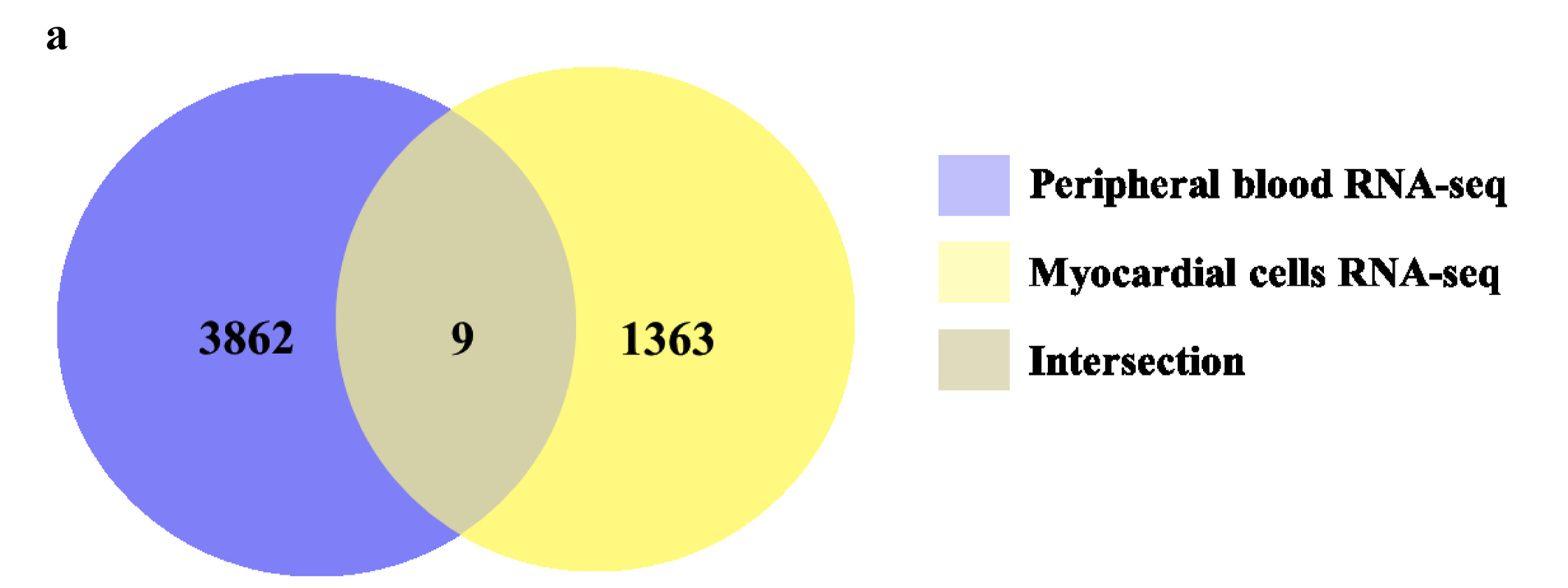

Supplement: Supplementary Figure 1 — circRNA screening. Totally 5 circRNAs were screened out after comparison of our results with human heart tissue sequencing results. [file Image_1.TIF]

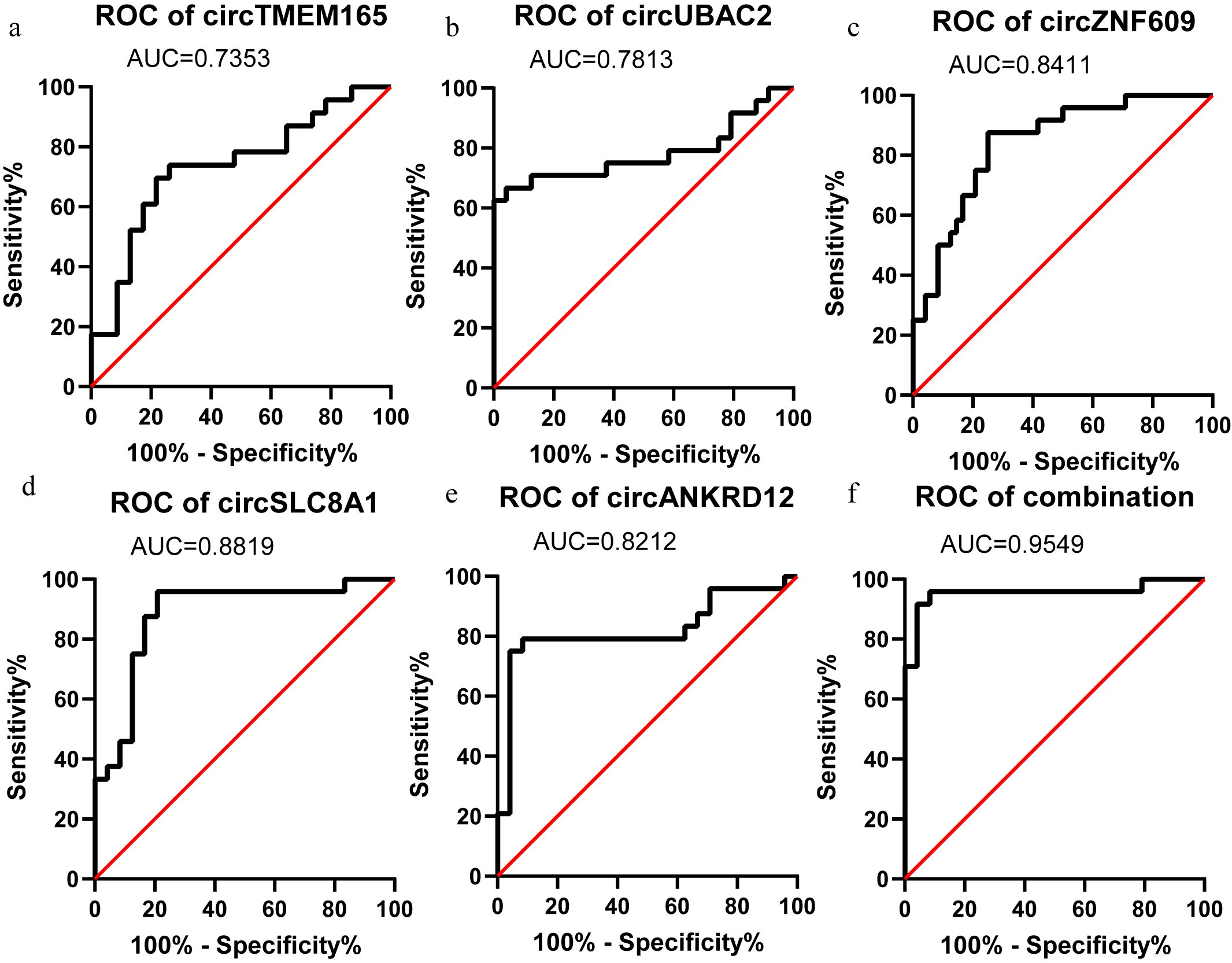

Supplement: Supplementary Figure 2 — ROC curve validation; 30% of the random sample was used to verify the ROC curve reliability of our data trained. [file Image_2.TIF]
